# Case report: change of dominant strain during dual SARS-CoV-2 infection

**DOI:** 10.1186/s12879-021-06664-w

**Published:** 2021-09-16

**Authors:** Andrei E. Samoilov, Valeriia V. Kaptelova, Anna Y. Bukharina, Olga Y. Shipulina, Elena V. Korneenko, Stepan S. Saenko, Alexander V. Lukyanov, Antonina A. Grishaeva, Antonina A. Ploskireva, Anna S. Speranskaya, Vasiliy G. Akimkin

**Affiliations:** Central Research Institute of Epidemiology of the Federal Service for Surveillance on Consumer Rights Protection and Human Wellbeing, 111123 Moscow, Russia

**Keywords:** SARS-CoV-2, COVID-19, Dual infection, High-throughput sequencing, Case report

## Abstract

**Background:**

The dual infection with SARS-CoV-2 is poorly described and is currently under discussion. We present a study of two strains of SARS-CoV-2 detected in the same patient during the same disease presentation.

**Case presentation:**

A patient in their 90 s was hospitalised with fever. Oropharyngeal swab obtained on the next day (sample 1) tested positive for SARS-CoV-2. Five days later, the patient was transferred to the ICU (intensive care unit) of the hospital specialising in the treatment of COVID-19 patients, where the patient's condition progressively worsened and continuous oxygen insufflation was required. Repeated oropharyngeal swab (sample 2), which was taken eight days after the first one, also tested positive for SARS-CoV-2. After 5 days of ICU treatment, the patient died. The cause of death was a coronavirus infection, which progressed unfavourably due to premorbid status. We have performed sequencing of full SARS-CoV-2 genomes from oropharyngeal swabs obtained eight days apart. Genomic analysis revealed the presence of two genetically distant SARS-CoV-2 strains in both swabs. Detected strains belong to different phylogenetic clades (GH and GR) and differ in seven nucleotide positions. The relative abundance of strains was 70% (GH) and 30% (GR) in the first swab, and 3% (GH) and 97% (GR) in the second swab.

**Conclusions:**

Our findings suggest that the patient was infected by two genetically distinct SARS-CoV-2 strains at the same time. One of the possible explanations is that the second infection was hospital-acquired. Change of the dominant strain ratio during disease manifestation could be explained by the advantage or higher virulence of the GR clade strain.

**Supplementary Information:**

The online version contains supplementary material available at 10.1186/s12879-021-06664-w.

## Background

Dual infection is a phenomenon where an individual is simultaneously infected with two or more strains of the same virus. It can affect host immune responses and result in increased fitness of the viral population. In recent years, a number of cases when individuals were infected with more than one strain of HIV have been identified [1–3]. The findings of dual infections have been reported for influenza viruses [4], the Epstein–Barr virus [5] and other viruses. Cases of SARS-CoV-2 reinfection are reported in the scientific literature [6–8]. Coinfection with respiratory pathogens [9–13], and other viruses, including (but not limited to) HIV (human immunodeficiency virus) [14, 15], Epstein–Barr virus [16], as well as bacterial and fungal confections [17, 18] in COVID-19 (coronavirus disease 2019) patients were also described, however, there are little to no reports of double infection with SARS-CoV-2 (severe acute respiratory syndrome coronavirus 2), except for in two works [19, 20]. Here, we present a case report of an individual with two genetically distinct SARS-CoV-2 strains during the same disease manifestation. These strains belonged to different phylogenetic clades: GH and GR. Our findings suggest that the relative abundance of the strains could change significantly over time.

## Case presentation

A patient in their 90 s with a history of chronic persistent atrial fibrillation, chronic heart failure and hypertension was hospitalised with fever (38 °C), with the admission diagnosis of lobar pneumonia, unspecified organism (J18.1). The patient denied travel or contact with persons with symptoms of COVID-19. Oropharyngeal swab obtained on the next day (sample 1) tested positive for SARS-CoV-2 (cycle threshold, Ct = 13, measured using AmpliSens® Cov-Bat-FL assay kit). Five days later, the patient was transferred to the ICU (intensive care unit) of the hospital specialising in the treatment of COVID-19 patients and prescribed Kaletra, Levofloxacin, Clexane, ACC and Aspirin. During the observation period, the patient's condition progressively worsened and continuous oxygen insufflation was required. Oxygen saturation ranged from 70% without oxygen support to 92–98% with oxygen support via a face mask (5 L/min). Repeated oropharyngeal swab (sample 2), which was taken eight days after the first one, also tested positive for SARS-CoV-2 with high viral load (Ct = 13, measured using AmpliSens® Cov-Bat-FL assay kit). After five days of ICU treatment, the patient died. The cause of death was a coronavirus infection, which progressed unfavourably due to premorbid status.

This research was approved by the local ethics committee of the Central Research Institute of Epidemiology of the Federal Service for Surveillance on Consumer Rights Protection and Human Wellbeing on 17.11.2020. Written informed consent was obtained from the patient for publication of this case report and any accompanying images. A copy of the written consent is available for review by the Editor-in-Chief of this journal.

We have performed the sequencing and bioinformatics analysis of two swab samples obtained from the same patient eight days apart using the SCV-2000 bp protocol and Illumina sequencing (see Additional file 1 “Methods” for more detailed description). Briefly, the sequencing of sample 1 yielded 1.1 M paired-end raw reads. After quality filtration and PCR primer trimming, 837 thousand reads remained, 99.87% of which were mapped to the reference SARS-CoV-2 genome strain hCoV-19/Wuhan/WIV04/2019 (MN996528.1). The mapping of trimmed reads to the reference sequence revealed seven heterogeneous positions (see Additional file 2: Fig. S1 for an illustration).

The sequencing of sample 2 yielded 3.9 M paired-end raw reads. After quality filtration and PCR primer trimming, 3.7 M reads remained, 99.93% of which were mapped to the reference SARS-CoV-2 genome strain hCoV-19/Wuhan/WIV04/2019 (MN996528.1). We analysed the mapped reads and found the same heterogeneity at the same positions as in Sample 1, but at a much lower level.

**Fig. 1:**
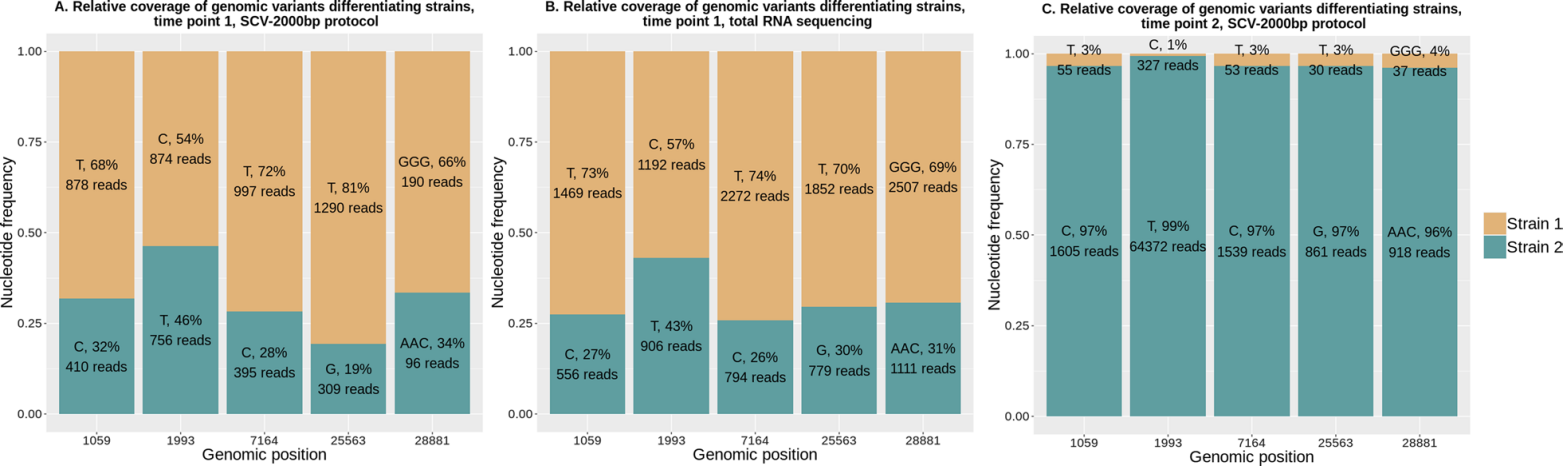
Relative coverage of SARS-CoV-2 genomic variants, which differentiate strains 1 and 2. Genomic positions are at the X-axis, their relative frequencies are at Y-axis. **A** Sample 1, sequencing using genome fragments amplification (SCV-2000 bp protocol); **B** Sample 1, sequencing using total RNA library; **C** Sample 2 (collected eight days later), sequencing using genome fragments amplification (SCV-2000 bp protocol)

We interpreted our observations as the simultaneous presence of two SARS-CoV-2 strains in the same patient’s samples. After obtaining consensus genomic sequence from the dominant strain from the less heterogeneous second sample (hereafter referred to as strain 2), it became possible to unambiguously reconstruct genomic sequences of the strain prevalent in the first sample (hereafter referred to as strain 1). The relative abundance of strains 1 and 2 in both time points was assessed by averaging the relative coverage of heterogeneous positions (Fig. 1A) and amounted to roughly 69% and 31% in the first sample and 3% and 97% in the second sample, respectively (Fig. 1C). We found that strain 1 was dominant in sample 1, and strain 2 became dominant in sample 2.

The resulting sequences are available at GenBank with accession numbers MW305251.1 (dominant strain from Sample 1) and MW305250.1 (the dominant strain from Sample 2, collected eight days after the first swab).

Heterogeneity in the sequence reads can also be explained by sequencing artifacts arising from the polymerase errors, chimeric fragments generation during nucleic acid amplification, and contamination during RNA extraction and library preparation. To exclude the possibility that the observed heterogeneity is a mistake, we isolated RNA from the original swabs for the second time and performed sequencing of libraries prepared from total RNA without any enrichment for both samples.

RNA-seq of sample 1 yielded 12.6 M paired-end 250 bp long reads. After quality filtration and PCR primer trimming, 12.2 M reads remained, 2.29% of which were mapped to the reference SARS-CoV-2 genome strain hCoV-19/Wuhan/WIV04/2019 (MN996528.1). We compared RNA-seq reads with reads obtained using the SCV-2000 bp protocol (Fig. 1B). Roughly the same frequencies of alternative nucleotides prove that observed heterogeneity was not a result of a sequencing artifact and that DNA originating from two different SARS-CoV-2 strains is present in sample 1. Read coverage at the genomic positions differentiating strain 1 from strain 2 varied from 2100 to 4090 in sample 1 after total RNA sequencing.

RNA-seq of Sample 2 yielded 15.0 M paired-end raw reads. After quality filtration and PCR primer trimming, 14.6 M reads remained, and only 0.04% of them were mapped to the reference SARS-CoV-2 genome strain hCoV-19/Wuhan/WIV04/2019 (MN996528.1), which was not enough to confirm the presence of a minor fraction of reads (about 3%) representing strain 1.

To confirm the presence of two different strains in the same sample, we have designed a set of 16 primers pairs (Additional file 3) aimed at the position of GGG28881AAC mutation (present in GR clade and absent in others, including GH). One of the primers from the pair overlaps one or two nucleotides at the positions 28,881–28,883 (either GGG or AAC) at the 3′ end, so every primer pair is expected to uniquely amplify the fragment of GR clade genome (8 primer pairs) or non-GR clade genome (8 primer pairs). We have performed PCR with all of the designed primers pairs with the cDNA from both samples, successfully obtained amplicons, and performed high-throughput sequencing. We have mapped paired reads to the reference sequence and discovered the GGG28881AAC mutation in the amplicons aimed at GR clade or absence of this mutation in the amplicons aimed at non-GR clade for both sample 1 and sample 2 with nearly 100% frequency for the most of the primer pairs (see Additional file 4: Fig. S2 for an illustration). This experiment confirms the presence of two different strains belonging to different GISAID clades in both samples.

Paired sequence reads were deposited to SRA with the accession number PRJNA719737 in the NCBI BioProject database, SAMN18616569 and SAMN18616570 stand for sample 1 and sample 2, respectively.

Comparison of strain 1 genomic sequence to all of the GISAID SARS-CoV-2 database (as of 11 November 2020) revealed that this sequence is unique to GISAID, the closest genomes having at least two mismatches compared to strain 1 genome. Out of 571 closest sequences, only three originated from Russia (EPI_ISL_428905, EPI_ISL_428875 and EPI_ISL_428871, all of them were collected in March), most of the other genomes originated from the USA (402), Iceland (28) and Canada (26) and were collected in March-early April.

Comparison of strain 2 genomic sequence to all of the GISAID SARS-CoV-2 database (as of 11 November 2020) revealed 1062 genomic sequences with 100% identity to strain 2, 78 out of which originated from different regions of Russia, with 10 of them collected in Moscow (collection dates of which vary from late March to early April), including eight genomes obtained in our lab, as described in [21]. The latest genomes with 100% identity to strain 2 were collected in Saint-Petersburg in the middle of September (EPI_ISL_602339 and EPI_ISL_602340). Other genomes with 100% identity to strain 2 originated mostly from England (333), Portugal (121) and the USA (86) and were collected mainly in March and April.

Comparison of strain 1 and strain 2 genomic sequences with the reference strain hCoV-19/Wuhan/WIV04/2019 (MN996528.1) revealed four nucleotide mutations present in both of them (C241T in the non-coding region; C3037T, a synonymous substitution in NSP3 protein; C14408T, resulting in P323L mutation in NSP12 protein; and A23403G, resulting in D614G mutation in spike protein), as well as four mutations present only in strain 1 (C1059T, resulting in T85I mutation in NSP2 protein; T1993C, a synonymous substitution in NSP2 protein; C7164T, resulting in T1482I mutation in NSP3 protein; and G25563T, resulting in Q57H in NS3 protein) and three mutations present only in strain 2 (G28881A and G28882A, resulting in R203K mutation in N protein; G28881C, resulting in G204R mutation in N protein) (Table 1).

**Table 1:** List of mutations present in strains 1 and 2

Reference (MN996528.1)				Strain 1 (MW305251.1)			Strain 2 (MW305250.1)		
Position	Nucleotide	Protein	Amino acid	Nucleotide	Amino acid	Mutation name	Nucleotide	Amino acid	Mutation name
241	C	–	–	T	–	–	T	–	–
1059	C	NSP2	T	**T**	**I**	**T85I**	C	T	–
1993	T	NSP2	Y	**C**	**Y**	**Synonymous substitution**	T	Y	Synonymous substitution
3037	C	NSP3	F	T	F	Synonymous substitution	T	F	Synonymous substitution
7164	C	NSP3	T	**T**	**I**	**T1482I**	C	T	–
14,408	C	NSP12	P	T	L	P323L	T	L	P323L
23,403	A	Spike	D	G	G	D614G	G	G	D614G
25,563	G	NS3	Q	**T**	**H**	**Q57H**	G	–	–
28,881	G	N	R	G	R	–	**A**	**K**	**R203K**
28,882	G	N	R	G	R	–	**A**	**K**	
28,883	G	N	G	G	G	–	**C**	**R**	**G204R**

Mutations differentiating strain 1 from strain 2 are highlighted in bold

**Fig. 2:**
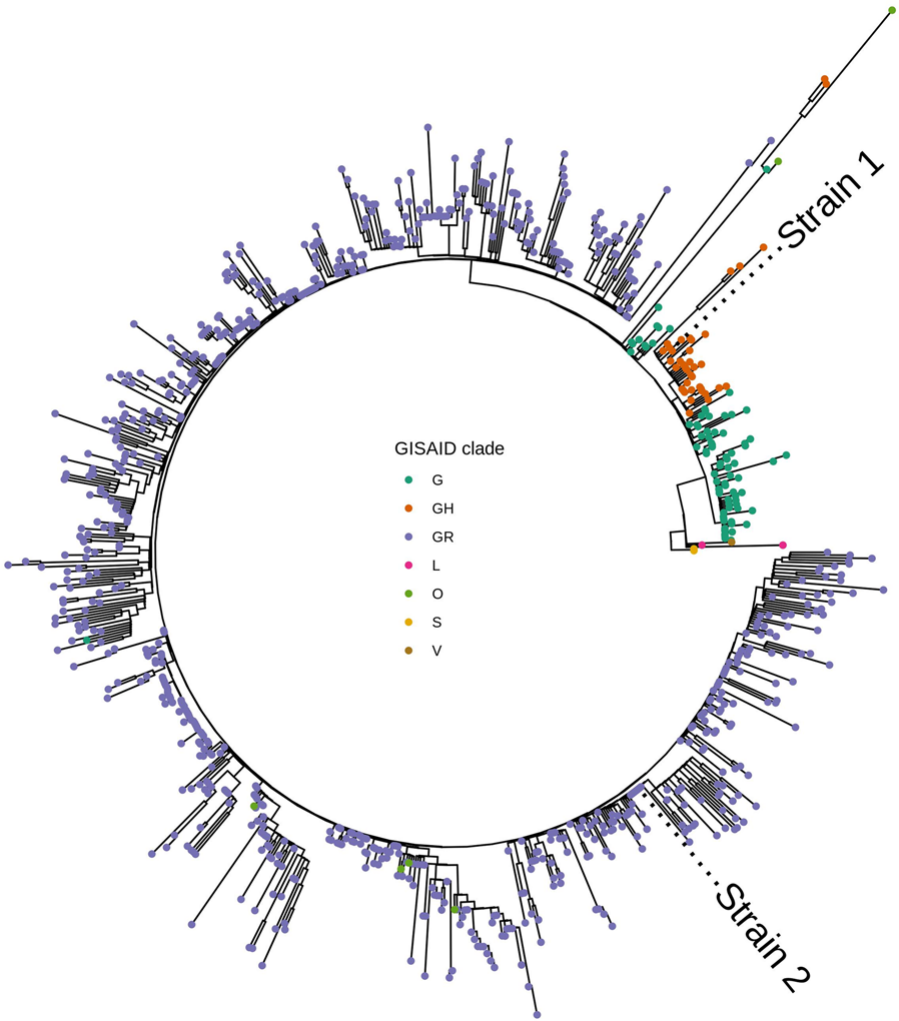
Phylogenetic tree of all of the available SARS-CoV-2 strains isolated in Russia (as of 11 November 2020). GISAID clade classification is represented by colour. Tip labels mark positions of strain 1 (MW305251.1) and strain 2 (MW305250.1). Closely related to SARS-CoV-2 virus strain bat/Yunnan/RmYN02/2019 (GISAID ID EPI_ISL_412977) was used as a root (not shown)

Phylogenetic analysis was performed by building a tree of all of the available SARS-CoV-2 genomes from the samples collected in Russia. It has been revealed that strain 1 (MW305251.1) belongs to the GH clade, and strain 2 (MW305250.1) belongs to the GR clade (GISAID classification) (see Fig. 2).

## Discussion and conclusions

Dual infection can affect host immune responses and result in increased fitness of the viral population. HIV dual infection contributes to rapid disease progression [1] and increased viral load [2], and requires antiretroviral treatment effective against both viruses [3]. Meanwhile, despite a rapidly growing body of evidence, there is little to no information about dual SARS-CoV-2 infection. To our knowledge, the possibility of SARS-CoV-2 double infection was discussed in two works [19, 20]. Authors of both provided no information about patients’ medical history and viral subpopulation dynamics during disease progression, and almost no information about clade classification of SARS-CoV-2 analysed strains.

Liu et al. [19] cite the CEO of CODE Genetics biopharmaceutical company Kari Stefansson, who has reported a patient hospitalized in Iceland infected by two SARS-CoV-2 subtypes simultaneously in early March 2020. One strain of the SARS-CoV-2 coronavirus was more aggressive, while the second strain is a mutation from the original version of the coronavirus that appeared in Wuhan, China. This was regarded as the first known case of co-infection. Authors have detected the presence of signature mutations from different phylogenetic groups in the same genomes and explained it as possible co-infection or homogenous recombination. However, it can also be explained as contamination or sequencing errors, and reliable confirmation of the authors' hypothesis requires a more in-depth analysis of obtained data.

Hashim et al. [20] utilized Sanger sequencing to obtain short (795 bp) fragments of spike protein gene. They have discovered double peaks and interpreted it as double infection in all 19 analysed samples, with most of the detected mutations being missense. The authors discussed that co-infecting strains could compensate for the damaging effect of the truncated spike protein. Our observations show different dynamics between two strains, where one was replaced with another after several days. In our opinion, the results of Hashim et al. need to be confirmed using high-throughput sequencing technology because the authors' interpretation of Sanger sequencing data is questionable. Most of the electropherograms presented in the manuscript [20] consist of double peaks with low intensity of the minor peak, which makes interpretation of the obtained data challenging. Minor variants can be reliably detected in the 20% mixture analysed trace in conventional Sanger sequencing; however, in the analysed traces from the 10% and 5% mixtures, the fragment is indistinguishable from the baseline noise [22].

The mutation rate of SARS-CoV-2 inside the same host is a critical parameter for understanding viral evolution because it may become more infectious or more virulent. Choi et al. [23] described the case of persistent infection (over 150 days) accompanied by accelerated evolution of SARS-CoV-2 in an immunocompromised patient. On days 18 and 25, sequencing of SARS-CoV-2 genomes obtained from the patient revealed five amino acid substitutions compared to the reference, but later their number grew to over 20. The largest number of mutations was detected in the Spike protein, especially in the receptor-binding domain. We have not observed the appearance of new mutations, but strain 1, discussed in this work, is closely related to the strain obtained from the immunocompromised patient on day 18 (both of them possess mutations Spike_D614G, NS3_Q57H, NSP2_T85I and NSP12_P323L, but differ in mutation NSP13_T115I present only in the strain obtained from the immunocompromised patient and mutation NSP3_T1482I present only in strain 1).

SARS-CoV-2 quasispecies and genetic diversity within the same individual or cell culture were discussed in several articles and preprints [24–28]. Low fidelity of RNA polymerases results in heterogeneity in the RNA virus population. Here, we report a dual infection because two strains we detected belong to different phylogenetic clades, which cannot be explained by RNA polymerase errors.

The presence of two viral variants in the same patient might be associated with nosocomial infection. However, the patient spent only one day in the hospital before the first swab was collected, in which we have detected two SARS-CoV-2 strains. The probability of getting a positive PCR test result in the early days after infection is rather low [29]. Therefore, our data can be interpreted either as an infection with two strains before admission to the hospital or as a rapid increase in hospital strain viral load to a detectable level due to the patient’s weakened immune system. The severity and rapid progression of the disease, along with unchangeably high viral load (Ct = 13), could be associated with either a change in the dominant strain of SARS-CoV-2 or the patient's elderly age [30].

Our results show a drastic change in both strains’ abundance: the dominant strain from the first sample almost disappeared in the sample obtained a week later. The strain dominating in the first sample belongs to the GH clade, while the strain which prevailed in the second sample belongs to the GR clade. Change of the dominant strain in the viral community can be a stochastic event, or it can be caused by many factors, including exposure to nosocomial infection, the individuality of the host immune response, or difference in strain fitness. Our experiment's design does not allow us to find out the cause of change in the viral community (we have analyzed only two samples taken from a single patient), but it allows us to detect mutations differentiating one strain from another and discuss their potential effect on strain fitness. A longitudinal study of similar cases with double infection can potentially shed light on the connection between mutations in the SARS-CoV-2 genome and change of strain abundance in the viral community.

In our case, potentially disadvantageous mutations (present in strain 1, which decreased its abundance over time) include Q57H in NS3 protein, T85I in NSP2 protein and T1482I in NSP3 protein. NS3_Q57H demarcates the GH clade [31]. As of 11 November 2020, it occurs in 4.6% (40 out of 874) of Russian SARS-CoV-2 sequences. Possible effects of this mutation were discussed in several papers. In a work by Gupta et al. [32], who used protein modelling, its effect was predicted as deleterious. Alam et al. discussed that it prevents ion permeability by constricting the channel pore more tightly, possibly reducing viral release and immune response [33]. In work by Wang et al. [34], the authors suggested that it can make the SARS-CoV-2 more infectious. The second mutation present only in strain 1, NSP2_T85I, is rare in Russia and occurs in 3.4% (30 out of 874) Russian SARS-CoV-2 genomes (as of 11 November 2020). This mutation also has predicted deleterious functional outcome [35]. Wang et al. [34] discussed that this mutation benefits from other mutations like Spike_D614G and NS3_Q57H and could enhance infectivity. Finally, NSP3_T1482I mutation is present in only 96 genomes submitted to GISAID (as of 11 November 2020), and, to our knowledge, it was never discussed in scientific literature.

Potentially advantageous mutations (present in the dominant strain in the second sample) include R203K and G204R in N protein. These mutations occur together as a result of the substitution of three consecutive nucleotides. The presence of these mutations demarcates the GR clade [31]. Over 85% of Russian SARS-CoV-2 genomes submitted to GISAID (as of 11 November 2020) belong to the GR clade and possess both of these mutations. According to different protein modelling approaches, these mutations either destabilise N protein [33] or have a neutral effect [32]. Several articles point out the association of this clade with higher mortality [36] or significant prevalence in severely ill or deceased patients and also higher prevalence in females and children compared to other clades [37].

Other mutations present in both strains are D614G in spike protein and P323L in NSP12. Spike_D614G is one of the most widely discussed mutations. Its presence demarcates the G clade [31], it increases infectivity [38–40] and mortality [41, 42], alters viral fitness [43], and, according to protein modelling, enhances the folding stability of the spike protein [34]. It is present in 99.2% of SARS-CoV-2 genomes obtained from Russia (as of 11 November 2020). NSP12_P323L is predicted to make the polymerase more rigid, which may increase the replication speed [34] and mutation rate [44, 45]. It is present in 97.9% of Russian SARS-CoV-2 genomes (as of 11 November 2020).

In our work, we present a case of dual SARS-CoV-2 infection, which allowed us to discuss the potential difference in the relative fitness of two genetically distant strains. The effect of SARS-CoV-2 dual infection on viral load and the severity or duration of COVID-19 is currently unknown. It is possible that the presence of two different SARS-CoV-2 strains was a factor which led to rapid progression of the patient’s disease and death. The importance of research aimed at cases similar to ours can hardly be overestimated because it provides insights into the molecular epidemiology of COVID-19 and can help detect potentially advantageous mutations which increase virulence and fitness of SARS-CoV-2.

Our study shows the case of dual SARS-CoV-2 infection by two phylogenetically distant strains and the viral community dynamics. Dominant strain from the first sample belonged to GH clade, while GR clade strain became dominant eight days after collecting the first sample.

## Supplementary Information

**Additional file 1.** Description of methods used for SARS-CoV-2 full genome sequencing and data analysis.

**Additional file 2: Figure S1.** IGV snapshot showing the heterogeneity in mapped Illumina reads in the first sample. 66% of the reads match the reference SARS-CoV-2 sequence strain hCoV-19/Wuhan/WIV04/2019 (MN996528.1), 34% of reads have GGG- > AAC substitution at positions 28,881–28,883.

**Additional file 3.** List of primer pairs used to confirm the presence of two viral strains from different clades in the same sample. One of the primers from the pair overlaps one or two nucleotides at the positions 28881–28883 (either GGG or AAC) at the 3′ end, so every primer pair is expected to uniquely amplify the fragment of GR clade genome with GGG28881AAC mutation (8 primer pairs with GR in the name of one of the primers) or non-GR clade genome (8 primer pairs with GH in the name of one of the primers).

**Additional file 4: Figure S2.** IGV snapshot showing the result of the sequencing of PCR products obtained using primers uniquely amplifying a fragment of viral genome with (two bottom libraries, GR clade) or without (two upper libraries, non-GR clade) GGG28881AAC mutation. All of the PCR products shown were amplified using cDNA from sample 2.

## Data Availability

Full-genome sequences of strain 1 and 2 are available at GenBank using accession numbers MW305251.1 and MW305250.1, respectively. Paired sequence reads were deposited to SRA with the accession number PRJNA719737 in the NCBI BioProject database.

## Abbreviations

cDNA: Complementary deoxyribonucleic acid
COVID-19: Coronavirus disease 2019
Ct: Cycle threshold
HIV: Human immunodeficiency virus
ICU: Intensive care unit
PCR: Polymerase chain reaction
RNA: Ribonucleic acid
SARS-CoV-2: Severe acute respiratory syndrome coronavirus 2
