# Supplementary material for: Case report: change of dominant strain during dual SARS-CoV-2 infection
Source: BMC Infect Dis. 2021 Sep 16;21:959. doi: 10.1186/s12879-021-06664-w (PMC8443909; doi:10.1186/s12879-021-06664-w)
Supplement: Supplementary file 1 — Additional file 1. Description of methods used for SARS-CoV-2 full genome sequencing and data analysis. [file 12879_2021_6664_MOESM1_ESM.docx]

Methods

RNA (ribonucleic acid) was extracted using the QIAamp Viral RNA Mini Kit (QIAGEN, Germany) according to the manufacturer’s instructions. Reverse transcription reaction was performed using 10 μL of the RNA samples, random hexanucleotide primers and Reverta-L kit (AmpliSens, Russia) according to the manufacturer’s instructions. Libraries for whole-genome sequencing of SARS-CoV-2 were prepared using single-strand cDNA (сomplementary deoxyribonucleic acid) and SCV-2000bp protocol described previously [1]. In brief, we amplified four pools of ~2000 bp long fragments covering the whole SARS-CoV-2 genome. The obtained PCR (polymerase chain reaction) products were purified, mixed equally and sheared in microTUBE-50 AFA Fiber Screw-Cap (PN 520166) using Covaris M220 (Covaris, Woburn, MA). Libraries were constructed using Y-shaped adapters compatible with Nextera XT Index Kit and amplified with 8 cycles using oligos from Nextera XT Index Kit and Q5 High-Fidelity DNA Polymerase (New England BioLabs, NEB). Libraries for sequencing of amplicons aimed at the position of GGG28881AAC mutation were prepared using the same protocol as described above without shearing.

Libraries for total RNA sequencing were prepared using the NEBNext® Ultra™ II Directional RNA Second Strand Synthesis Module E7550 (New England BioLabs, NEB) according to the manufacturer’s instructions. Double-stranded cDNA was sheared in microTUBE-50 AFA Fiber Screw-Cap (PN 520166) using Covaris M220 (Covaris, Woburn, MA) using the following settings: peak incident power — 75W, duty factor — 10%, cycles per burst — 200, treatment time — 40 s, temperature — 20 °C and sample volume — 50 µl. Libraries were constructed with NEBNext® Ultra™ II End Repair/dA-Tailing Module (E7546), NEBNext Ultra II Ligation Module (E7595) (New England BioLabs, NEB) according to the manufacturer’s instructions. Amplification of libraries was performed with Q5 High-Fidelity DNA Polymerase (M0491) using NEBNext® Multiplex Oligos for Illumina® (Index Primers Set 2, E7500) (New England BioLabs, NEB) according to the manufacturer’s instructions, 25 μL in total with 10 cycles of amplification.

Size selection of the libraries was performed using Agencourt AMPure XP (Beckman Coulter, Danvers, MA, USA). Quality and fragment length distribution of the obtained libraries were evaluated with Agilent Bioanalyzer 2100 (Agilent Technologies, USA). Sequencing was performed on Illumina HiSeq 1500 with HiSeq PE Rapid Cluster Kit v2 and HiSeq Rapid SBS Kit v2 (500 cycles).

Raw reads from amplicon libraries and total RNA libraries were processed as described in [21]. We performed adapter and quality trimming with Trimmomatic [2] using parameters SLIDINGWINDOW:4:25 MINLEN:40 ILLUMINACLIP:2:20:10, removed non-internal PCR primer sequences with cutadapt (for amplicon libraries) [3] and mapped the reads to the reference sequence (strain hCoV-19/Wuhan/WIV04/2019, MN996528.1, GISAID accession ID EPI_ISL_402124) using bowtie2 [4] with the parameter --local. After that, we filtered out reads with low mapping quality (less than 9) using SAMtools [5] and reads with soft-clipped sequence during alignment, performed base-calling with GATK HaplotypeCaller using parameter -ploidy 1 [6] and filtered gvcf files with default parameters of BCFtools [7]. Finally, the consensus sequences were obtained with BEDTools consensus [8]. The validity of the resulting sequence was verified manually by visual inspection of mapped reads. Areas with coverage lower than 50 were masked with NNN. Nucleotide frequency for mutations differentiating strains from each other was determined by analysing allelic depths (AD) after variant calling with GATK Mutect2. Relative abundance of strains was assessed as a mean value of allelic frequencies at the genomic positions differentiating strains from each other. All of the SARS-CoV-2 strains were aligned using mafft [9], the phylogenetic tree was built with IQtree 2 [10] using the GTR model.

References

1. Speranskaya AS, Kaptelova V V, Valdokhina A V, Bulanenko VP, Samoilov AE, Korneenko E V et al. SCV-2000bp: a primer panel for SARS-CoV-2 full-genome sequencing. bioRxiv 2020; : 2020.08.04.234880.
2. Bolger AM, Lohse M, Usadel B. Trimmomatic: a flexible trimmer for Illumina sequence data. Bioinformatics 2014; 30: 2114–2120.
3. Martin M. Cutadapt removes adapter sequences from high-throughput sequencing reads. EMBnet.journal 2011; 17: 10.
4. Langmead B, Salzberg SL. Fast gapped-read alignment with Bowtie 2. Nat Methods 2012; 9: 357–359.
5. Handsaker B, Wysoker A, Fennell T, Ruan J, Homer N et al. The Sequence Alignment/Map format and SAMtools. Bioinformatics 2009; 25: 2078–2079.
6. McKenna A, Hanna M, Banks E, Sivachenko A, Cibulskis K, Kernytsky A et al. The Genome Analysis Toolkit: A MapReduce framework for analyzing next-generation DNA sequencing data. Genome Res 2010; 20: 1297–1303.
7. Danecek P, McCarthy SA. BCFtools/csq: haplotype-aware variant consequences. Bioinformatics 2017; 33: 2037–2039.
8. Quinlan AR, Hall IM. BEDTools: a flexible suite of utilities for comparing genomic features. Bioinformatics 2010; 26: 841–842.
9. Katoh K. MAFFT: a novel method for rapid multiple sequence alignment based on fast Fourier transform. Nucleic Acids Res 2002; 30: 3059–3066.
10. Minh BQ, Schmidt HA, Chernomor O, Schrempf D, Woodhams MD, von Haeseler A et al. IQ-TREE 2: New Models and Efficient Methods for Phylogenetic Inference in the Genomic Era. Mol Biol Evol 2020; 37: 1530–1534.
