# Supplementary figures and images for: Case report: change of dominant strain during dual SARS-CoV-2 infection
Source: BMC Infect Dis. 2021 Sep 16;21:959. doi: 10.1186/s12879-021-06664-w (PMC8443909; doi:10.1186/s12879-021-06664-w)

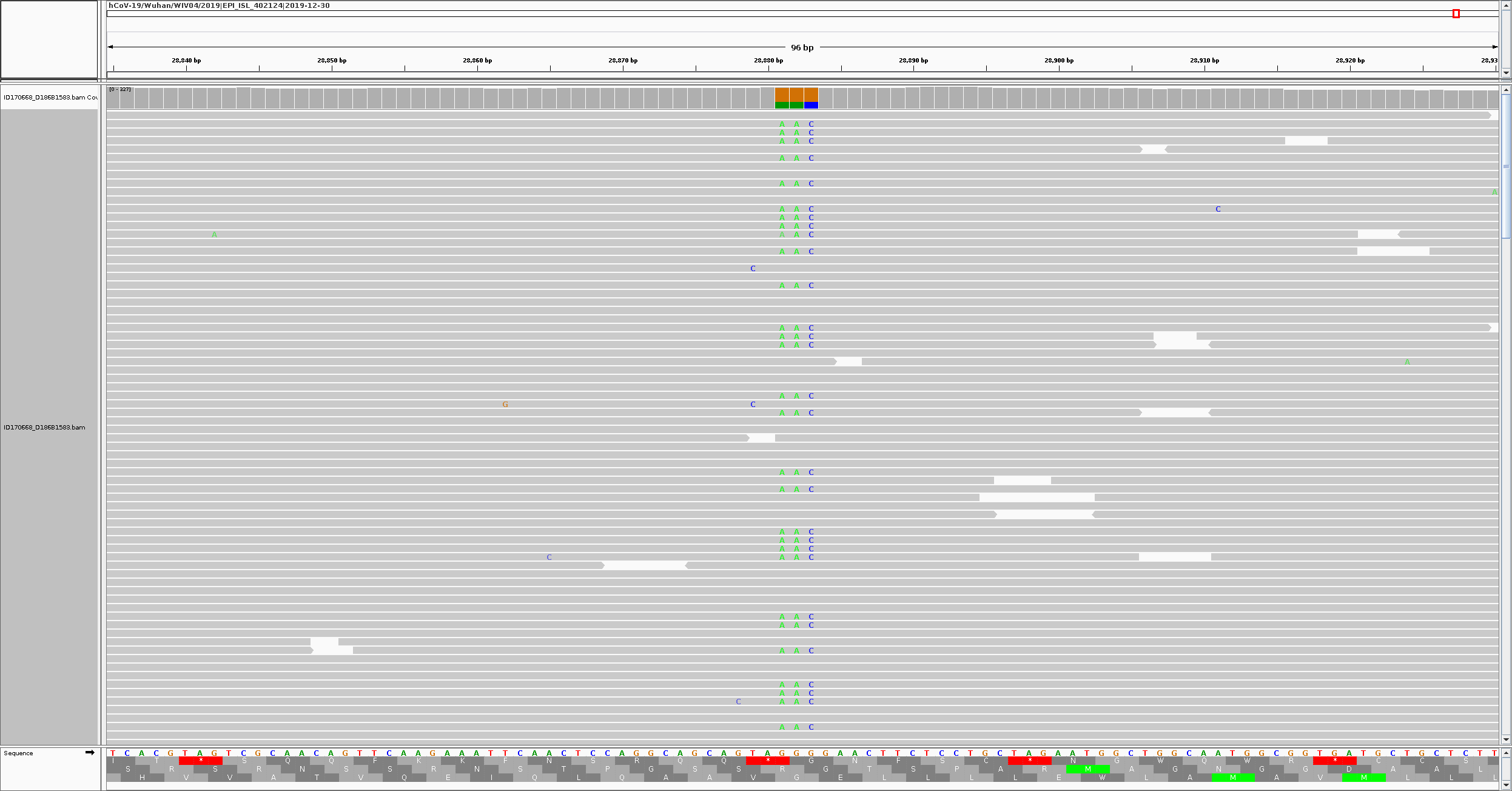

Supplement: Supplementary file 2 — Additional file 2: Figure S1. IGV snapshot showing the heterogeneity in mapped Illumina reads in the first sample. 66% of the reads match the reference SARS-CoV-2 sequence strain hCoV-19/Wuhan/WIV04/2019 (MN996528.1), 34% of reads have GGG- > AAC substitution at positions 28,881–28,883. [file 12879_2021_6664_MOESM2_ESM.tif]

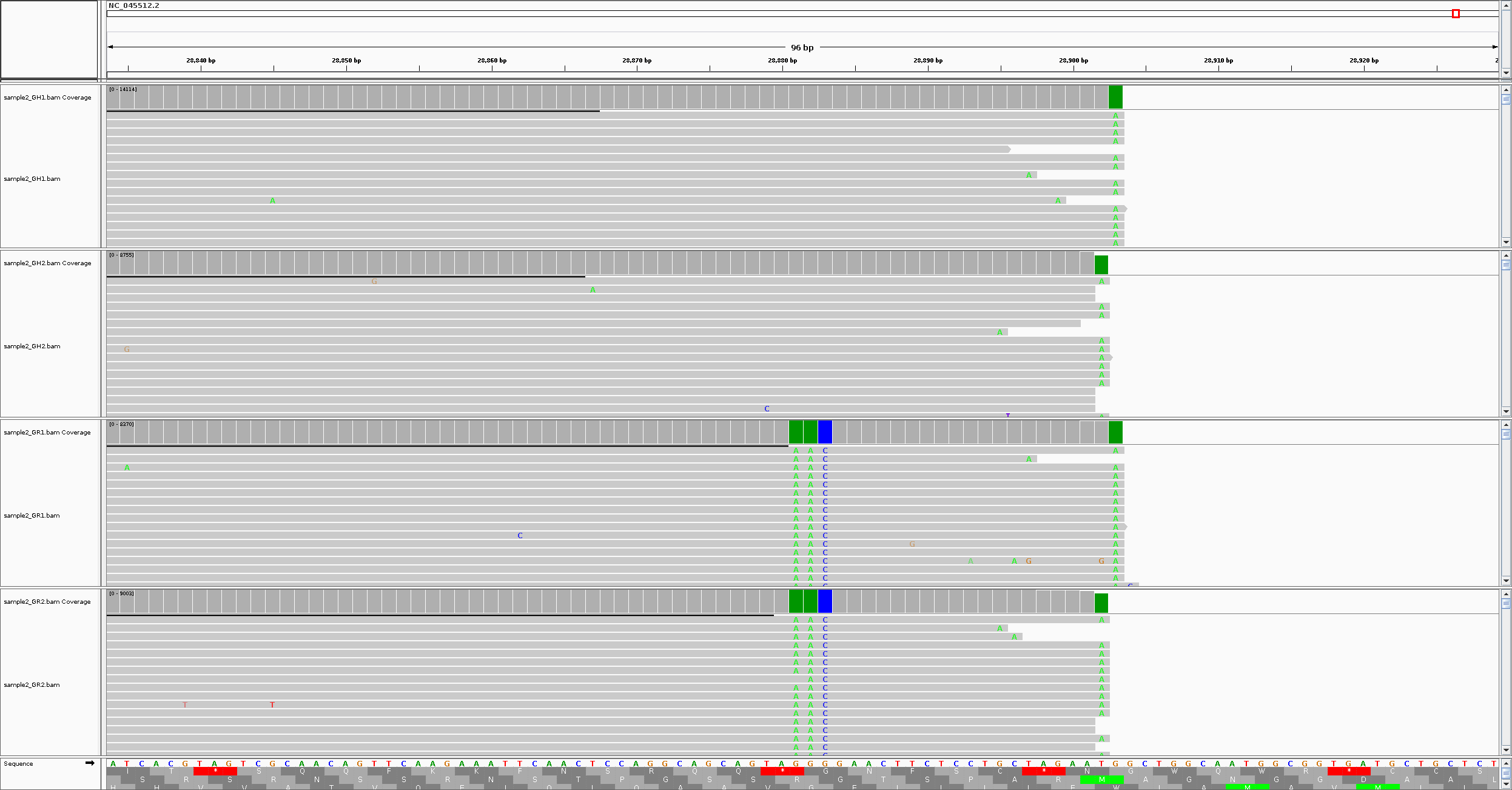

Supplement: Supplementary file 4 — Additional file 4: Figure S2. IGV snapshot showing the result of the sequencing of PCR products obtained using primers uniquely amplifying a fragment of viral genome with (two bottom libraries, GR clade) or without (two upper libraries, non-GR clade) GGG28881AAC mutation. All of the PCR products shown were amplified using cDNA from sample 2. [file 12879_2021_6664_MOESM4_ESM.png]
